# Between- and within-Group Differences in Fruit and Vegetable Purchases, Consumption, and BMI among Hispanic Farmers’ Market Shoppers Who Use SNAP

**DOI:** 10.3390/ijerph18189923

**Published:** 2021-09-21

**Authors:** Ginnie Sawyer-Morris, Sara Grajeda, Tara Tracy, Allison Karpyn

**Affiliations:** 1 Human Development & Family Sciences, University of Delaware, Newark, DE 19716, USA; gsmorris@udel.edu; 2 Center for Research in Education and Social Policy, University of Delaware, Newark, DE 19716, USA; sbchap@udel.edu (S.G.); tetracy@udel.edu (T.T.)

**Keywords:** body mass index, Hispanic, Latino, farmers markets, fruit and vegetable purchasing, fruit and vegetable consumption, supplemental nutrition assistance program (SNAP)

## Abstract

(1) Background: Despite considerable efforts to increase farmers’ market access (FM) and improve household fruit and vegetable (FV) purchasing in low-income communities, little is known about the FV purchasing and consumption characteristics of low-income Hispanic farmers’ market shoppers. (2) Methods: A secondary analysis of baseline data from a farmers’ market study conducted between 2015 and 2017 (*n* = 2825) was performed. Participants who also received supplemental nutrition assistance program (SNAP) completed a 31-item online survey assessing demographics, health characteristics, and FV purchasing and consumption habits. Descriptive statistics and bivariate analyses were used to assess between- and within-group differences amongst Hispanic and non-Hispanic households. Regression analyses were used to examine associations among BMI, FV purchasing and consumption, and household size for Hispanic and non-Hispanic households as well as for Hispanic subgroups. (3) Results: The sample included 515 Hispanic and 2310 non-Hispanic SNAP-using FM shoppers in 13 states. Despite experiencing significantly higher food insecurity (89% vs. 81%, non-Hispanic), Hispanic shoppers consumed similar amounts of FV (3.04 cups/day) and spent less doing so. Significant subgroup differences were identified for FV purchasing. (4) Conclusions: Findings emphasize the importance of food insecurity and household size in FV interventions and underscore the capacity of Hispanic families to maintain FV consumption.

## 1. Introduction

Rapid growth in immigration over the past fifty years has precipitated changes in the racial and ethnic composition of the United States population [1]. Fifty years ago, individuals of Hispanic ethnicity made up only 5% of the population whereas, today, over 60 million Hispanics live in the U.S. and comprise more than 18% of the total U.S. population [1,2]. As the U.S. Hispanic population grew, health disparities also increased. Rates of obesity and food insecurity are now higher among ethnic and racial minority groups while consumption of fruits and vegetables (FVs) is persistently lower than recommendations [3]. Next to non-Hispanic black adults (49.6%), Hispanic and Latin adults (44.8%) have the highest rates of obesity relative to other subgroups including non-Hispanic white adults (42.2%) and non-Hispanic Asian adults (17.4%) [4]. Rates of food insecurity are similarly higher for Hispanic households (15.6%) as compared to White, non-Hispanic households (7.9%) [5] and, during COVID-19, food insecurity in Hispanic households has increased to 47% [6].

Disparities in health are, in part, attributable to differences in food access where low-income residents often experience limited availability of affordable, high quality food within close proximity to their home [7]. As a result, families are left with more expensive, less healthy, and lower-quality options compared to the more affordable retail options available to higher-income White, non-Hispanic communities [8,9].

An increasingly popular approach to addressing disparities in food access in the U.S. is the establishment of farmers’ markets (FMs) that accept Supplemental Nutrition Assistance Program (SNAP) benefits. The steady growth in FMs, overall (1755 in 1994 to 8140 in 2019) [10], has been paralleled by increased SNAP spending at FMs, which climbed from $2.7 million in 2008 to more than $22.4 million in 2017 [11]. SNAP is a federal program that helps millions of low-income American families purchase healthy food and move toward self-sufficiency; each month, SNAP benefits are distributed on an electronic benefits transfer (EBT) card that functions as a debit card at SNAP-authorized retailers [12].

Access is not the only barrier to food security. Purchasing and consuming the recommended amount of FVs per day (two cups fruit, three cups vegetables depending on age and sex) [13] can require a substantial proportion of household income, making FVs unaffordable in many low-income communities [14]. Farmers’ markets have become an important context for public health interventions aiming to increase the affordability of FVs for low-income populations, including SNAP recipients. Since 2012, when additional funding was made available to expand SNAP redemption capabilities at FMs, a wide range of efforts to promote low-income families’ FV purchases at FMs have been undertaken including subsidy-type programs designed to increase the amount of money SNAP participants can spend at FMs [15]. In order to inform policies that increase the affordability of FVs for all SNAP participants, it is critical to gain an understanding of the racial/ethnic differences in SNAP- and health-related characteristics.

Research examining racial/ethnic differences in low-income FM shoppers is limited [16]. In their recent study of Illinois FM incentive program users, Singleton et al. discussed key racial/ethnic differences between non-Hispanic Black and non-Hispanic White shoppers, but due to low enrollment of Hispanic participants, the authors were unable to make robust comparisons of Hispanic participants across race or within Hispanic ethnicity [17]. This representation problem is not uncommon. SNAP serves a diverse population, yet studies evaluating purchasing habits and dietary quality of SNAP participants often overrepresent White participants or focus mainly on differences between non-Hispanic Black vs. non-Hispanic White shoppers [18]. The current study addresses this gap in two ways: first, by describing the potentially unique characteristics of low-income Hispanic FM shoppers in the US, and second, by examining differences between Hispanic v. non-Hispanic shoppers as well as exploring within-group differences in characteristics amongst Hispanic ethnicity groups (e.g., Mexican-American, Puerto-Rican, Cuban).

It is expected that Hispanics will differ significantly from non-Hispanics in terms of body mass index (BMI), food insecurity, and FV consumption. Hispanic families experience higher rates of obesity (44.8%) and food insecurity (15.6%) compared to non-Hispanic households [4]. However, in terms of FV intake, Hispanic adults demonstrate significantly higher prevalence of meeting the recommended guidelines compared to other race/ethnicity groups (i.e., 15.7% Hispanic vs. 14.3% non-Hispanic Black vs. 11.2% non-Hispanic White [reference]) [19]. However, findings are mixed and some research shows that these rates differ depending on region, gender, income level, and SNAP-participation status [20].

The Hispanic population in the U.S. is not a monolith, but a highly heterogeneous group with rich, ethnic-specific traditions, especially when it comes to food culture [21]. In order to design programming that is culturally responsive and tailored to meet the needs of the U.S. Hispanic and Latino community, it is important to build an evidence base that takes these group-specific variations into account. A number of studies investigating differences in vegetable consumption across Hispanic subgroups demonstrate that Hispanic origin and nativity make a difference in predicting type and frequency of consumption [22,23]. For example, Siega-Riz et al. found that total vegetable intakes differed among Cubans, Dominicans, and Puerto Ricans, and intakes of dark green/yellow and non-starchy vegetables differed between Puerto Rican and non-Puerto Rican Latina women [23]. In addition, in their study of nutrient and food intake during pregnancy, Hromi-Fiedler et al. found that Puerto Rican women who were more acculturated consumed higher levels of processed food and trans fatty acids than non-Puerto Rican Latinas [24]. Singleton et al. shed light on this gap in their intersectional review of U.S. consumer food purchasing, calling for future studies to conduct robust assessments of within-group differences in FV purchasing across ethnicities [25]. However, research on differences in FV purchasing and consumption amongst Hispanic subgroups remains limited, and to the authors’ knowledge, no studies to date have examined within-group differences in FV purchasing and consumption amongst Hispanic SNAP-using FM shoppers. The current study’s exploratory within-group analysis of FV purchasing, consumption, and BMI across Hispanic subgroups contributes evidence to this gap in the literature. Hispanics and Latinos represent a key constituency of SNAP participants (22.3%) and despite the growing body of literature on the benefits of FMs for SNAP-users, little is known about the unique characteristics of Hispanic shoppers [26]. The purpose of the current study is to characterize the Hispanic SNAP-using FM shopper based on SNAP- and health-related characteristics relevant to informing programming and policy. Findings are intended to contextualize and inform future FM interventions seeking to improve FV consumption, and overall health, among Hispanic shoppers.

## 2. Materials and Methods

Participants were recruited from 77 local FM in 13 states and were eligible for participation if they used SNAP-EBT at the FM. Data collection occurred between September 2015 and October 2017 as part of a larger study of shopping behavior and incentive use at FMs; the data presented in this paper examine baseline data from the larger study.

### 2.1. Participants and Recruitment

FMs that participated in the current study were from a national network operated by Wholesome Wave Inc., which provides staffing as well as technical and financial support to FMs at the state-, regional-, and individual-levels. All participating markets served both non-SNAP and SNAP-eligible customers, though the present study was limited to SNAP patrons.

SNAP-using FM shoppers were recruited on-site by market managers. If a FM shopper used SNAP-EBT, the market manager informed the shopper of the study and inquired whether they would be interested in participating. If so, the FM shopper provided their name, email address and/or cell phone number to the market manager. After each market session, the market manager sent the shoppers’ contact information to the research team who in turn sent the FM SNAP shoppers an electronic link with enrollment materials including a survey for the study via email or text. Enrollment could take place during any month of the study. Each data collection period lasted approximately four to six months and occurred in alignment with the growing season and/or FM schedule at that location. Upon survey completion, shoppers were provided a FM incentive of at least $0.40 per $1 SNAP spent and no more than $2 for every $1 SNAP spent to spend on FV, depending on FM standards.

This study and its procedures were approved by the University of Delaware Institutional Review Board (IRBNet ID# 748161). Informed consent was obtained from all participants and the survey included a statement regarding the details of the research and voluntary participation.

### 2.2. Measures

An online survey was administered in Spanish or English using QualtricsTM and consisted of 31 core questions (see Appendix A) that assessed demographic and household characteristics, food insecurity, FV purchasing and consumption, and BMI. Questions capturing demographic and household characteristics (e.g., gender, age, race, ethnicity, family size, income [NY only], and region) were based on U.S. Census parameters. Due to the terms of our partnership agreement with the national FM network, only New York (NY) FM shoppers were asked to report annual income. Federal eligibility for SNAP is limited to people with gross incomes up to 130% of the federal poverty line; and, given all participants in the current sample were enrolled in SNAP, SNAP was substituted as a proxy for low-income status.

Food insecurity was measured using the Hunger Vital Sign, a validated two-item food insecurity screener [27]. Overall health and health conditions were assessed using two questions from the Behavioral Risk Factor Surveillance System Questionnaire (BRFSS), a health-related survey sponsored by the Centers for Disease Control and Prevention for targeting and building health promotion activities [28]. Participants were also asked to report their height and weight measurements, which were used to calculate BMI and then interpreted using standard weight status categories [29].

The 10 FV questions from the DSQ assessed frequency of consumption of selected FVs within the past month including 100% fruit juice, fresh fruit, salads, other vegetables, potatoes, beans, and tomatoes [30,31]. The DSQ offers nine response categories, which range from “Never” to “2 or more times per day.” The National Health and Nutrition Examination Survey standard scoring algorithms were used to convert responses to estimates of participants’ daily FV consumption in cup equivalents [31]. Food purchasing estimates were obtained from responses to the questions “On average, how much do you spend per month for food and drinks to consume at home?” and “How much of this is spent on FVs (including fresh, frozen or canned items)?”Additionally, questions from the two-item food insecurity screener [27] asked participants to respond to the following statement, “Within the past 12 months we worried whether our food would run out before we got money to buy more,” and “Within the past 12 months the food we bought just didn’t last and we didn’t have money to get more.” Participants were considered food insecure if they answered “Sometimes” or “Often” to either question.

### 2.3. Statistical Analysis

Cases were deleted pairwise; if a participant skipped one question, they were excluded from analysis for the skipped question but were still included in the analyses of other questions with complete information. In addition, “unsure” or “prefer not to answer” responses were coded as missing. Outliers were defined as data points larger than the absolute value of 1.5 times the interquartile range and were recoded as missing values (<0.1%).

Basic descriptive and frequency statistics were calculated for continuous outcomes (i.e., FV purchasing, FV consumption, BMI) and categorical and continuous predictors (i.e., gender, age, annual income [NY participants only], geographical region, primary shopper status, food insecurity, household size; see Table 1). Between- and within-group differences in categorical predictors and Hispanic ethnicity were examined using chi-square tests. A negative binomial regression was used to examine the relationship between the number of people per household (i.e., household size) and Hispanic ethnicity. A negative binomial was chosen over a Poisson because the distribution of household size (number of people per household) was over-dispersed, meaning the variance (*s*^2^ = 3.35) was larger than the mean (*µ* = 2.81); overdispersion was further indicated by the significant dispersion parameter in the negative binomial model, alpha = 0.04, CI [0.025, 0.06]. To examine differences in FV consumption, purchasing, and BMI between Hispanic and non-Hispanic participants, independent sample T-tests were conducted for each outcome. Because three T-tests were conducted, a Bonferonni adjustment was used to reduce the alpha threshold for statistical significance to 0.017. To detect differences amongst Hispanic cultural subgroups (i.e., Mexican, Puerto Rican, Cuban, and Other) in each outcome, a one-way ANOVA with a Tukey post-hoc test was utilized.

Ordinary least squares regression was used to predict FV consumption, FV purchasing, and BMI based on food insecurity, household size, ethnicity, gender, age, geographical region, and obesity indicators. To allow for substantive interpretation, household size was only included in the model predicting purchasing, whereas obesity was included only in the model predicting consumption. Statistical Analysis System (SAS) 9.4 was used for all data management and analysis. Models were run separately for Hispanics and non-Hispanics. The Hispanic participant model included categorizations of Hispanic participants into cultural subgroups (i.e., Mexican, Puerto Rican, Cuban, and Other) to account for potential differences between subgroups. Due to the skewed nature of monthly FV expenses, FV purchasing, the dependent variable, was transformed using the natural log. Results from these models are therefore interpretable (after a reverse transformation) as percent change in FV purchasing based on a one-unit change in predictors.

## 3. Results

In total, 5186 potential participants in 13 states and the District of Columbia, nested within three regions (Northeast: New Hampshire, New York, Rhode Island, Vermont, District of Columbia, Maine; South: Florida, Virginia, Georgia, Louisiana; West/Midwest: Arizona, Missouri, Nevada, Ohio), provided accurate contact information to market managers. Of these, 3073 participants (59%) ultimately enrolled in the study and completed the online survey during six data collection periods held between 2015 and 2017. Baseline survey data included 515 Hispanic and 2310 non-Hispanic participants (*n* = 2825).

The Hispanic participants were majority female (90%), nearly 40% of Hispanic participants were between the ages of 28 to 37, and over 70% lived in the Northeast. The largest ethnic subgroup of Hispanic participants was Mexican or Mexican-American (33%). An additional 17% of the 515 Hispanic participants were Puerto Rican, 7% were Cuban, and 44% identified as another Hispanic ethnic group.

Overall, most Hispanic participants identified as being the primary food shopper (90%) in their household, and 4% stated they split the responsibility. Food insecurity was experienced by 89% of Hispanic participants in the last 12 months, and the average household size for Hispanic participants was 3.5 people. In terms of FV purchasing and consumption, Hispanic participants spent an average of $183 per month on FV and consumed an average of three cups of FV per day. The average BMI among Hispanic FM SNAP shoppers was 28.2 (SD = 6.6). The majority of Hispanic participants (64%) were overweight or obese, while 34% were in the normal weight category [29].

Food insecurity was more common among Hispanic participants (χ2 = 18.24, *p* < 0.001). Eighty-nine percent of Hispanics were considered food insecure in comparison with 81% of non-Hispanics. Additionally, while high rates of food insecurity were observed across all Hispanic subgroups, no statistically significant differences were found between them (χ2 = 5.99, *p* = 0.11).

Household size among Hispanic participants was predicted to be 1.35 times larger compared to non-Hispanic households, 95% CI [1.28, 1.4]. Amongst Hispanic subgroups, Mexican and Mexican-American households were predicted to be 1.41 times larger than Puerto Rican households, 95% CI [1.23, 1.62]; 1.39 times larger than Cuban households, 95% CI [0.13, 0.53]; and 1.37 times larger than households that identified as another Hispanic ethnicity, 95% CI [1.24, 1.52].

Since household sizes were larger, on average, for Hispanics, differences in mean FV purchases each month were compared based on purchases per person, calculated by dividing household purchase totals by the reported number of persons in the household. Hispanics reported spending $66.98 on FVs per person per month while non-Hispanic participants reported spending $75.11, a difference that was statistically significant (*t* = 3.07, *p* = 0.002). Hispanics therefore reported spending less on FVs per person while maintaining equivalent FV consumption. Within Hispanic subgroups, statistically significant differences also exist for monthly FV purchases per person (F = 7.13, *p* < 0.001) with Cubans spending the most at $87.69 per person. On the other end of the spectrum, Mexicans spent, on average, the least at $52.68 per person. Puerto Ricans spent an average of $66.65, and other Hispanics spent approximately $73.86 per person per month on FVs. Statistically significant differences were found between Cubans and Mexicans, and between Mexicans and other Hispanics.

Hispanic participants consumed an average of 3.04 cups of FVs per day while non-Hispanic participants consumed an average of 3.00 cups per day, a difference that was not statistically significant *t*(2256) = −0.67, *p* = 0.604). No within-group differences were found for Hispanic subgroups in terms of FV consumption.

On average, Hispanic participants had a BMI of 28.2. In comparison, non-Hispanic participants had a BMI, on average, of 28.7. BMI differences were not statistically significant between Hispanics and non-Hispanics *t*(2817) = 1.09, *p* = 0.28); however, within Hispanics, there were significant differences between subgroups (F = 3.35, *p* = 0.02). Both Puerto Ricans (average BMI of 29.41) and Mexicans (average BMI of 29.00) were statistically different from other Hispanic participants (average BMI of 27.25). Cubans had an average BMI of 27.97.

As shown in Table 2, food insecurity, household size, and obesity were each predictors of FV purchasing for Hispanics (F = 4.99, *p* < 0.001, R^2^ = 0.140). Each additional member of the household was associated with a 10% increase in FV purchasing. Hispanic participants who were food insecure spent 21% more than those who were not food insecure. Finally, Hispanic participants who were obese spent 15% less on FV compared to Hispanics who were not obese. For non-Hispanic participants, household size, age groups 28–37 and 38–47, and obesity were significant predictors of FV purchasing (F = 33.88, *p* < 0.001, R^2^ = 0.157); food insecurity was not a significant predictor. With each increase in non-Hispanic household size, FV purchasing increased 16%. Non-Hispanic participants ages 28–37 purchased 17% more FV and those ages 38–47 purchased 18% more.

The model predicting FV consumption for Hispanic participants was not significant. For non-Hispanics, however, gender, age group 38–47 (vs. 18–27), region, and obesity were significant predictors of FV consumption (F = 8.02, *p* < 0.001, R^2^ = 0.037). Female non-Hispanics and individuals with obesity consumed less FV, whereas individuals between the ages of 38–47 consumed more FV (see Table 2).

For both Hispanic BMI (F = 3.91, *p* < 0.001, R^2^ = 0.088) and non-Hispanic BMI (F = 15.31, *p* < 0.001, R^2^ = 0.060), gender, age, and the Midwest/West region were positively associated with BMI. Food insecurity, however, was only a significant predictor of BMI for non-Hispanics. There were no significant differences between Hispanic subgroups for any of the regression models.

## 4. Discussion

SNAP is a diverse population and despite growth in the US Hispanic population among key ethnic subgroups [2,26], Hispanic populations remain underrepresented in studies examining FM shopping- and health-related behaviors [16,17]. This study highlighted key characteristics of Hispanic SNAP-using FM shoppers as well as differences between Hispanic and non-Hispanic households and within Hispanic ethnicities. Findings revealed that Hispanic families were larger (*M* = 3.5), more food insecure (89%), and spent more of their monthly grocery budget on FV ($182.52) compared to non-Hispanic households. Within-group findings showed that the Mexican/Mexican-American families were larger and spent significantly less per person on FV compared to Cuban and Other Hispanic households; and Mexican/Mexican-American and Puerto Rican participants’ BMIs were significantly higher than participants who identified as another Hispanic ethnicity. Furthermore, both Hispanic and non-Hispanic families’ FV purchasing decisions were influenced, in part, by household size and obesity; however, Hispanic families with food insecurity spent 20% more on FV compared to those who were not and this trend was not observed in the non-Hispanic model. Hispanics’ FV consumption was not associated with any of the predictors; however, non-Hispanics’ FV consumption was positively associated with being older (i.e., between ages 28–47), and negatively associated with being female and with obesity. Both Hispanic and non-Hispanic BMIs were positively associated (i.e,. BMIs were higher) with being female, with mid- to older-adulthood (i.e., age groups 48–57 and 58–67), and with living in the Midwest or West region. Unlike non-Hispanic households, Hispanic families’ BMI was not related to food insecurity, nor to age ranges 28–37 or 38–47. As such, findings suggest that Hispanic BMI among FM shoppers using SNAP may be less influenced by food insecurity relative to non-Hispanic households.

Results indicate that household sizes were larger overall in Hispanic families, a trend that was the most evident for Mexican/Mexican-American households, and Hispanic families who use SNAP at FMs demonstrate unique FV purchasing patterns compared to other ethnicities. Namely, Hispanic families consume the same amount of FVs while spending less; this phenomenon was all the more true for Hispanics who were food insecure. The specific drivers of these differences cannot be determined based on data available in this study; however, prior literature on Hispanic health outcomes posits these differences are likely related to socioeconomic and socio-cultural factors (e.g., acculturation, nativity) [32,33]. Accordingly, future research into the magnitude of influence of other moderators and mediators, both quantitatively and qualitatively, would help to shed light on these findings.

### 4.1. Key Findings

Our study found that Hispanics were more food insecure (89%) than other groups (81%), yet consumed similar amounts of FV (3.04 cups/day vs. (3.00 cups/day, non-Hispanic) and spent less doing so. While the average FV consumption rates captured by the current study remain below the national guidelines (three cups of vegetables and two cups of fruit per day, depending on one’s age and sex) [13], Hispanic participants demonstrated a unique ability to maintain similar levels of FV consumption compared to other groups while spending less, demonstrating a potential resilience in relation to food insecurity.

Future studies should consider whether this trend holds true in larger samples with study designs that include culturally-relevant variables such as acculturation and nativity, alongside other important factors such as income and family size. Previous studies have shown that Hispanic families in the U.S. maintain lower mortality rates relative to non-Hispanic whites despite lower education levels and lower socioeconomic status; this phenomenon is referred to as the “epidemiological paradox,” or “Hispanic paradox” [34]. Future studies could explore whether the “Hispanic paradox” extends to Hispanic SNAP-using FM shoppers’ purchasing and nutrition-related behaviors. Such investigations could build on the current study’s between- and within-group analyses by longitudinally examining whether shopping and nutrition-related behaviors act as mediators and/or moderators of mortality rates for Hispanic vs. non-Hispanic SNAP-using FM shoppers as well as within Hispanic subgroups. Further, given the current findings, future studies exploring the explanatory mechanisms of the “Hispanic paradox” could consider including household size as an additional mediator or moderator of Hispanic mortality rates.

In the U.S., the average size of Hispanic households is 3.25 people compared with 2.43 for the total population [35]. This trend held true for Hispanics in our study, but we also found within group differences related to household size. Mexicans tended to have larger households than both Cubans and Puerto Ricans. The tendency for Hispanics to have larger households could be due to a greater prevalence of extended families among Hispanics compared to non-Hispanic whites, wherein six to 10 percent of family households within Hispanic subgroups are extended, relative to three percent of non-Hispanic white family households [1].

After adjusting for demographics, no significant findings were observed for Hispanic FV consumption. Prior research on FV consumption among Hispanics who use SNAP is limited; and, findings in the broader literature are mixed. A review by Ayala et al. showed that Hispanic families who are less acculturated consume fruit, rice, beans, and less sugar and sugar-sweetened beverages [33]; this association was substantiated in more recent studies of Hispanic FV intake [22,24]. Further, DiNoia found that non-Hispanic Whites consumed greater amounts of vegetables from the “Other” category in the 2011–2015 BRFSS (e.g., tomatoes, tomato juice or V-8 juice, corn, eggplant, peas, lettuce, cabbage, and white potatoes that are not fried such as baked or mashed potatoes) than Hispanics and that FV intake type differs by Hispanic culture of origin [22]. The variation across studies substantiates the need for the inclusion of culturally relevant variables that provide explanatory power to findings related to Hispanic households (e.g., acculturation, nativity, race/culture of origin).

### 4.2. Implications for Research

The largest Hispanic ethnicity group in our study was the Other (*n* = 224) category, comprising 43.5% of the overall Hispanic participant group (*n* = 515). We observed a number of significant findings specific to the “Other” category, however, were unable to make any meaningful interpretations due to lack of explanatory power. This limitation has critical measurement implications for future research. The Hispanic ethnicity question used in our study was based on the 2010 U.S. Census Bureau Hispanic origin question; however, due to initial concerns about survey length and open-ended response processing, our survey did not include a space for participants who identified as another Hispanic ethnicity to write in their country/culture of origin. This proved to be problematic as many Hispanic individuals prefer to self-identify using their specific ancestry as opposed to the general category “Hispanic or Latino” [36], a term not used outside the United States [37]. Furthermore, recent evaluation studies of racial/ethnic survey items from the U.S. Census indicate that people are more likely to report Hispanic ethnicity if the ethnicity item precedes the race item and includes an ethnicity category with which the respondent self-identifies [38].

Moving forward, we recommend that future studies not only examine program impacts and needs with more nuance, including country of origin but also provide a write-in option for individuals who identify as “Hispanic or Latino, Other” to report their ancestry, culture, or country of origin (see example in Appendix B).

Despite our study ending three years ago, the food insecurity rates reported for Hispanic participants in our sample are higher than the rates reported by Schanzenbach & Pitts for Hispanic families during COVID-19 (i.e., 47%) [6]. This discrepancy may be due to a difference in the way food insecurity was measured. For example, a recent study examining the association of FV subsidies with food purchasing among low-income communities used the same food insecurity screener (DSQ’s two-item food insecurity screener) as that used in the current study and subsequently reported comparable rates of food insecurity (i.e., 91.7%) to those reported for our sample (89%, Hispanic; 81% non-Hispanic) [39]. The vast differences in the rate of food insecurity reported by Schanzenbach & Pitts compared to both our study and Berkowitz et al.’s belies the importance of future research adhering to strict reporting standards in terms of measurement [6,39].

### 4.3. Implications for Policy and Practice

On 16 August 2021, the USDA distributed a press release describing modernizations of the Thrifty Food Plan, a cost-effective guide for purchasing nutritious foods to be prepared at home for a family of four [40]. The Thrifty Food Plan is used to inform monthly SNAP allotment. The expedited rollout of the re-evaluation was in response to President Biden’s January 2021 executive order delineating actions for an all-of-government effort to provide economic relief for American families during the COVID-19 crisis [41]. Among these actions was a call for the USDA to update food assistance benefits to reflect the true cost of a basic healthy diet. As a result of the re-evaluation, monthly SNAP benefits have been increased to $36.24 per person, effective 1 October 2021. In the midst of COVID-19, this expansion represents important progress towards closing the food insecurity gap for individuals enrolled in SNAP as well as an unprecedented opportunity to better understand how the program can best serve ethnic subgroups.

Our study also has implications for FM voucher programs, specifically. Findings showed that household size and food insecurity were both key predictors of FV purchasing for Hispanic FM shoppers who, when compared to non-Hispanics, reported larger households and were more likely to spend a larger proportion of their monthly grocery budget on FV even after adjusting for food insecurity. The “Agriculture Improvement Act of 2018 (H.R. 2)” (i.e., the Farm Bill) authorized the Gus Schumacher Nutrition Incentive Program (GusNIP) program to receive Congressional and Presidential priority for increasing SNAP beneficiaries’ ability to purchase FVs [42]. We suggest that future FV incentivizing programs take these findings into account by, for example, adding an additional incentive amount for each person in the household. Efforts to subsidize FVs and other healthy foods through programs like GusNIP, the Special Supplemental Nutrition Program for Women, Infants, and Children (WIC), the Farmers Market Incentive Program (FMNP), and the Senior FMNP should consider how to move from a benefit that offers one household amount to benefits that can be adjusted for household size (including revising maximum caps on established incentive increases). Including household size in future policies may be one mechanism of better serving Hispanic families who are more likely to have larger households and support extended family structures.

Further, we hope our study raises new questions about how to approach nutrition education programming among Hispanic SNAP participants. For example, community-based programs and nutrition education efforts, such as the Supplemental Nutrition Assistance Program Education (SNAP-Ed), could consider incorporating nutrition education tailored to meet the needs of Hispanic families in addition to broad based educational efforts related to the importance of FV consumption. Prior work in this area may also inform future considerations. For example, Otero-Sabogal et al. has recommended different nutrition education approaches based on acculturation and has found that educational messages targeting less acculturated Hispanics should encourage maintenance of healthy consumption practices (e.g., eating fruits, rice, beans) and focus on decreasing fat consumption, while for more acculturated Hispanics, placing emphasis on revisiting traditional cultural practices as they relate to food and diet may be more beneficial [43].

### 4.4. Limitations

While this study provides perspective on the unique characteristics that influence Hispanic families’ FV purchasing, FV consumption, and BMI, it has several limitations. Our sample was specific to SNAP shoppers at FMs; the results are therefore limited to this population. However, evidence from the current study builds on similar findings in other settings related to higher FV consumption among Hispanics. The comparison with similar non-Hispanic participants strengthens the theory that Hispanics are likely to have different food behaviors and health metrics based on cultural backgrounds. Furthermore, ours was a convenience sample of SNAP participants who shopped at FMs, which limits the generalizability of our results. Our use of an online survey may have discouraged potential participants with limited access and/or technological literacy. While this final limitation is worth noting, anecdotal evidence from market managers suggests that few SNAP shoppers were unable to participate due to lack of an email address or cell phone. Future research should consider adopting rigorous designs (e.g., random control) to maximize generalizability of findings.

Additionally, FV consumption and BMI were measured only for the primary shopper in the home. It is unlikely that FV consumption or BMI were the same for all members of the household. Evidence shows that mothers who are food insecure are more likely to make trade-offs to preserve their children’s food security by eating whatever is left over or by skipping meals entirely [44]. By capturing BMI and FV consumption of all family members, future studies will gain a better understanding of how the primary shoppers’ nutrition- and health-related behaviors differ from other members of their household.

FV purchasing is self-reported, and little is known about how Hispanic study participants may report finances differently than non-Hispanics, if at all. A report from the Teachers Insurance and Annuity Association of America (TIAA) Institute examining financial literacy amongst Hispanic individuals found that nativity made a difference; Hispanic individuals who were born in the U.S. demonstrated better financial literacy, demonstrated by their ability to accurately report finances, compared to those who were born outside of the U.S. [45]. Future studies could consider including nativity in models examining FV purchasing.

As mentioned previously, the sample sizes of the Hispanic subgroups are small and potential differences between subgroups may not be detectable as a result. In addition, only a select number of nationality subgroups were used in this study. A larger Hispanic sample containing additional subgroups may demonstrate differing patterns of FV consumption, purchasing, and BMI. It is recommended that future studies oversample Hispanic participants and incorporate the aforementioned recommendations for measuring ethnicity (see Appendix B).

Emergency and institutional food are two key sources of FV consumption for low-income populations. Because neither of these sources were the focus of the larger FM study, we were unable to explore this connection; however, future studies could collect information about these contexts in order to control for confounding effects.

A number of other culturally-relevant controls were not available in the current study including income, poverty rate, nativity, and acculturation. Poverty rates are high among Hispanic ethnicities, and while SNAP use served as a proxy for low-income in this study, it would have been more informative to know whether level of income or level of poverty was associated with any of the outcomes. Last, we recognize that prior research has explored nativity (e.g., US-born) and acculturation (e.g., first-generation, second-generation) as explanatory characteristics for differences in FV purchasing and consumption among Hispanics [22,46,47,48,49], however such data were not available for this study. Future studies should consider including survey items that capture these characteristics.

## 5. Conclusions

While many interventions targeting FV consumption at FMs are intended to improve access for low-income residents, they have not been tailored to serve our nation’s growing Hispanic population and the diversity of subcultures it comprises. The current study contributed evidence regarding differences in FV purchasing, consumption, and BMI between Hispanic and non-Hispanic SNAP-using FM shoppers, as well as within-group differences amongst Hispanic ethnicities. Findings may prove useful to researchers and policy-makers seeking to evaluate the effectiveness of FV incentive programming at FMs for low-income Hispanic families. Future research studies examining racial/ethnic differences with this population should include a write-in option for ethnicity/culture of origin survey items (see Appendix B) and consider including variables that are culturally relevant to Hispanic populations, including acculturation and nativity, as they may enhance explanatory power of findings. Future policy efforts to subsidize FV for low-income populations should consider taking into account household size as it may serve as an important mechanism in promoting FV purchasing and consumption for Hispanic families who use SNAP.

## Figures and Tables

**Table 1:** Descriptive Statistics and Between- and Within-group Comparisons for Hispanics vs. non-Hispanics and Hispanic Subgroups.

	Between-Group			Within-Group (Hispanic)				
Variable	Non- Hispanic	Hispanic	*p*-Value	Mexican- American	Puerto Rican	Cuban	Other	*p*-Value
*n*	2310	515		168	87	36	224	
Participants	82%	18%		6%	3%	1%	8%	
Female	**81.3%**	**89.7%**	**<0.001**	**96.4%**	**87.4%**	**77.8%**	**87.4%**	**0.002**
Age								
18–27	15%	16.2%	0.471	16.9%	19.8%	25.0%	13.0%	0.205
28–37	**26.2%**	**38.9%**	**<0.001**	**54.2%**	**30.2%**	**30.6%**	**32.3%**	**<0.001**
38–47	19.8%	23.7%	0.052	23.5%	16.3%	13.9%	28.3%	0.069
48–57	**16%**	**12.5%**	**0.049**	**4.8%**	**22.1%**	**16.7%**	**13.9%**	**0.001**
58–67	**16.4%**	**5.3%**	**<0.001**	**0.6%**	**8.1%**	**5.6%**	**7.6%**	**0.011**
68+	**6.6%**	**3.3%**	**0.005**	**0%**	**3.5%**	**8.3%**	**4.9%**	**0.016**
Annual Income ^†^								
<$15,000	56.0%	50.2%	0.147	**42.7%**	**54.9%**	**85.7%**	**51.9%**	**0.003**
$15,000–$25,000	29.3%	35.5%	0.101	47.2%	23.5%	0%	33.7%	0.126
$25,000+	14.7%	14.3%	0.908	10.1%	21.6%	14.3%	28.3%	0.083
Region								
Northeast	**46.8%**	**70.7%**	**<0.001**	**79.2%**	**77.0%**	**19.4%**	**70.1%**	**<0.001**
Midwest	**15.4%**	**2.1%**	**<0.001**	2.4%	1.2%	0%	2.7%	0.670
South	**32.6%**	**21.4%**	**<0.001**	**4.2%**	**19.5%**	**80.6%**	**25.5%**	**<0.001**
West	5.1%	5.8%	0.509	**14.3%**	**2.3%**	**0%**	**1.8%**	**<0.001**
Primary Shopper	90.9%	90.1%	0.565	88.7%	92.0%	94.4%	89.7%	0.681
Food Insecurity	**80.7%**	**88.7%**	**<0.001**	90.2%	82.4%	83.3%	91.0%	0.112
Household Size	**2.6**	**3.5**	**<0.001**	**4.3 ^‡^**	3.1 **	3.1 **	3.2 **	**<0.001**
FV Purchasing ($/month)	**146.98**	**182.52**	**<0.001**	$195.68	$165.55	$178.32	$180.73	N/S
FV Consumption (cups/day)	3.0	3.0	0.604	3.1	3.1	3.1	3.0	N/S
Average BMI	28.7	28.2	0.275	29.0 *	29.4 *	28.0	**27.3 ^‡^**	0.048

Note. *n* = 2825. Bold terms indicate significance at the 0.05-level. Primary shopper represents the proportion of participants that identified as the primary shopper in their respective household. Number of participants, gender (female vs. male), age, annual income [New York residents], geographical region, primary shopper, and food insecurity are reported as percentages of the total sample for the Hispanic v. non-Hispanic groups, and separately for Hispanic subgroups. Number of individuals (*n*), average household size, average fruit and vegetable (FV) purchasing and consumption, and average Body Mass Index (BMI) are reported numerically. All values are rounded to the first decimal position for convenient presentation. Chi-square tests were used to examine between- and within-group differences for categorical outcomes (i.e., female, age, income, region, primary shopper, food insecurity) for Hispanic vs. non-Hispanic ethnicities and Hispanic subgroups, respectively. Negative binomial regression was used to examine the relationship between- Hispanic vs. non-Hispanic ethnicities and within Hispanic subgroups and household size (number of persons). Independent samples t-tests with a Bonferroni adjustment (*p* < 0.017) were used to examine between-group differences in continuous outcomes (i.e., FV purchasing, FV consumption, BMI). A one-way analysis of variance (ANOVA) with a Tukey post hoc comparison was used to examine within-group differences. ^†^ Annual income was asked only of participants residing in New York. ^‡^ Reference category. N/S = Overall model not significant. ** p < 0.001, * *p* < 0.05.

**Table 2:** Regression Analyses Predicting Fruit and Vegetable Purchasing, Consumption, and BMI in Hispanic and non-Hispanic groups.

Estimates (Std. Err.)	FV Purchasing		FV Consumption (Cups)		Body Mass Index (BMI)	
	Hispanic	Non-Hispanic	Hispanic	Non-Hispanic	Hispanic	Non-Hispanic
Household Size	0.100 *** (0.021)	0.151 *** (0.011)	--	--	--	--
Food Insecurity	0.207 * (0.098)	0.025 (0.037)	0.138 (0.150)	0.070 (0.057)	−0.179 (0.916)	1.672 *** (0.427)
Female	0.171 (0.108)	0.046 (0.038)	−0.160 (0.163)	−0.298 *** (0.058)	2.696 ** (0.957)	2.283 *** (0.434)
Age						
18–27 ^†^	--	--	--	--	--	--
28–37	0.116 (0.096)	0.153 ** (0.048)	0.132 (0.146)	0.131 (0.072)	1.091 (0.878)	1.575 ** (0.542)
38–47	0.152 (0.103)	0.164 ** (0.050)	0.194 (0.158)	0.236 ** (0.076)	1.750 (0.950)	2.972 *** (0.572)
48–57	−0.103 (0.119)	0.027 (0.052)	−0.046 (0.183)	0.002 (080)	3.671 ** (1.102)	1.968 ** (0.601)
58–67	0.116 (0.156)	−0.013 (0.051)	0.332 (0.243)	−0.040 (0.079)	3.532 ** (1.457)	1.960 ** (0.596)
68 and above	−0.076 (0.179)	−0.045 (0.067)	0.147 (0.283)	−0.068 (0.103)	−0.423 (1.729)	1.146 (0.785)
Region						
Northeast ^†^	--	--	--	--	--	--
South	−0.060 (0.084)	0.014 (0.033)	−0.117 (0.130)	0.107 * (0.051)	−1.046 (0.792)	−0.414 (0.381)
Midwest/West	0.041 (0.125)	−0.013 (0.039)	0.294 (0.189)	−0.046 (0.060)	3.855 (1.144) **	2.905 *** (0.446)
Obese	−0.159 ** (0.067)	−0.215 *** (0.031)	0.007 (1.05)	−0.223 *** (0.048)	--	--
Hispanic Subgroups						
Mexican ^†^	--	--	--	--	--	--
Puerto Rican	−0.065 (0.098)	--	0.735 (0.551)	--	0.983 (0.910)	--
Cuban	0.109 (0.145)	--	−0.236 (0.829)	--	0.889 (1.368)	--
Other	0.028 (0.080)	--	−0.018 (0.438)	--	−1.038 (0.721)	--
Constant	4.355 *** (0.239)	4.368 *** (0.059)	2.942 *** (0.244)	3.192 *** (0.088)	24.603 *** (1.464)	23.327 *** (0.667)
Overall Model						
R^2^	0.140	0.157	0.023	0.037	0.088	0.060
F	4.99	33.88	0.84	8.02	3.91	15.31
*p*-value	<0.001	<0.001	0.613	<0.001	<0.001	<0.001

Note. Models were run separately for Hispanic and non-Hispanic groups. Bold terms indicate significance at the 0.05-level. ^†^ Indicates reference category. Due to the skewed nature of monthly fruit and vegetable (FV) expenses, FV purchasing, the dependent variable, was transformed using the natural log. Results from these models are therefore interpretable (after a reverse transformation) as percent change in FV purchasing based on a one-unit change in predictors. *** *p* < 0.001, ** *p* < 0.01, * *p* < 0.05.

## Data Availability

The data presented in this study are not publicly available currently due to privacy restrictions for human subjects participants.

## Appendix A

SNAP Shopper Survey

Welcome to the SNAP Incentive Lottery & Survey

Thank you for participating in the Farmers’ Market shopper survey. This short survey will take about 5 min to complete and will help us understand your experiences with how you shop for food and what you eat. Your answers are very important to us, so please read each question carefully and answer as best you can. After getting a little background information about you, we will ask questions about the foods you bought, ate, or drank during the past month (30 days). When answering, please include meals and snacks at home, restaurants, and anyplace else. The survey is short—it will take only about 5 min to complete. At the end of the survey, you’ll find out whether you have won the lottery for bonus money to help purchase fruits and vegetables, or to receive a free, re-usable grocery bag. Three out of four survey participants will win something. Even you don’t win, you’ll still receive the market bonus offered to all SNAP shoppers at the market. AND, there’ll be another opportunity to re-enter the lottery next month! This survey is part of a research study funded by Wholesome Wave and the USDA that is being conducted by the Center for Research in Education and Social Policy (CRESP) at the University of Delaware. All responses will remain confidential. Your participation in this study is voluntary. You are welcome to exit the survey at any time. For more information about this study and your rights as a participant please visit http://www.cresp.udel.edu/fini/participants/.

Please select “I agree” below if you would like to participate in this study and enter the lottery for a chance to win extra incentives toward the purchase of fruits and vegetables.

❍ I agree

1. At which farmers market did you complete a fruit & vegetable lottery ticket?
2. Are you the person who does most of the shopping for food in your household?

❍ Yes
❍ No
❍ I split it equally with another household member
❍ I don’t know
❍ Prefer not to answer

3. On average, how much do you spend per month for food and drinks to consume at home?

Expenses in Dollars

4. How much of this is spent on fruits and vegetables (including fresh, frozen or canned items)?

Expenses in Dollars

5. How many people does it feed?

[Questions 6-10 were asked under certain regional/seasonal criteria:]

6. Answer If At which farmers market did you complete a fruit & vegetable lottery ticket?-ME Is Selected Or At which farmers market did you complete a fruit & vegetable lottery ticket?-NH Is Selected Or At which farmers market did you complete a fruit & vegetable lottery ticket?-RI Is Selected Or At which farmers market did you complete a fruit & vegetable lottery ticket?-VT Is Selected
  Over the past month, how much would you say you spent on the following produce items?
  Apples
  Blueberries
  Carrots
  Peppers
7. Answer If At which farmers market did you complete a fruit & vegetable lottery ticket?-DC Is Selected
  Over the past month, how much would you say you spent on the following produce items?
  Greens
  Melons
  Nectarines
  Peppers
8. Answer If At which farmers market did you complete a fruit & vegetable lottery ticket?-MO Is Selected Or At which farmers market did you complete a fruit & vegetable lottery ticket?-OH Is Selected
  Over the past month, how much would you say you spent on the following produce items?
  Grapes
  Potatoes
  Melons
  Peaches
9. Answer If At which farmers market did you complete a fruit & vegetable lottery ticket?-GA Is Selected Or At which farmers market did you complete a fruit & vegetable lottery ticket?-LA Is Selected Or At which farmers market did you complete a fruit & vegetable lottery ticket?-VA Is Selected
  Over the past month, how much would you say you spent on the following produce items?
  Blueberries
  Cantaloupe
  Corn
  Figs
10. Answer If At which farmers market did you complete a fruit & vegetable lottery ticket?-AZ Is Selected Or At which farmers market did you complete a fruit & vegetable lottery ticket?-NV Is Selected
  Over the past month, how much would you say you spent on the following produce items?
  Beans
  Corn
  Melons
  Squash
11. Next, we would like to know a bit more about the foods you ate in the past month. During the past month, how often did you drink 100% pure fruit juices such as orange, mango, apple, grape and pineapple juices? Do NOT include fruit-flavored drinks with added sugar or fruit juice you made at home and added sugar to.

❍ Never
❍ 1 time last month
❍ 2–3 times last month
❍ 1 time per week
❍ 2 times per week
❍ 3–4 times per week
❍ 5–6 times per week
❍ 1 time per day
❍ 2 -3 times per day
❍ 4–5 times per day
❍ 6 or more times per day

12. During the past month, how often did you eat fruit? Include fresh, frozen or canned fruit. Do not include juices.

❍ Never
❍ 1 time last month
❍ 2–3 times last month
❍ 1 time per week
❍ 2 times per week
❍ 3–4 times per week
❍ 5–6 times per week
❍ 1 time per day
❍ 2 or more times per day

13. During the past month, how often did you eat a green leafy or lettuce salad, with or without other vegetables?

❍ Never
❍ 1 time last month
❍ 2–3 times last month
❍ 1 time per week
❍ 2 times per week
❍ 3–4 times per week
❍ 5–6 times per week
❍ 1 time per day
❍ 2 or more times per day

14. During the past month, how often did you eat any kind of fried potatoes, including french fries, home fries, or hash brown potatoes?

❍ Never
❍ 1 time last month
❍ 2–3 times last month
❍ 1 time per week
❍ 2 times per week
❍ 3–4 times per week
❍ 5–6 times per week
❍ 1 time per day
❍ 2 or more times per day

15. During the past month, how often did you eat any other kind of potatoes, such as baked, boiled, mashed potatoes, sweet potatoes, or potato salad?

❍ Never
❍ 1 time last month
❍ 2–3 times last month
❍ 1 time per week
❍ 2 times per week
❍ 3–4 times per week
❍ 5–6 times per week
❍ 1 time per day
❍ 2 or more times per day

16. During the past month, how often did you eat refried beans, baked beans, beans in soup, pork and beans, or any other type of cooked dried beans? Do NOT include green beans.

❍ Never
❍ 1 time last month
❍ 2–3 times last month
❍ 1 time per week
❍ 2 times per week
❍ 3–4 times per week
❍ 5–6 times per week
❍ 1 time per day
❍ 2 or more times per day

17. During the past month, NOT including what you answered about green salads, potatoes, or beans, how often did you eat other vegetables?

❍ Never
❍ 1 time last month
❍ 2–3 times last month
❍ 1 time per week
❍ 2 times per week
❍ 3–4 times per week
❍ 5–6 times per week
❍ 1 time per day
❍ 2 or more times per day

18. During the past month, how often did you have Mexican-type salsa made with tomato?

❍ Never
❍ 1 time last month
❍ 2–3 times last month
❍ 1 time per week
❍ 2 times per week
❍ 3–4 times per week
❍ 5–6 times per week
❍ 1 time per day
❍ 2 or more times per day

19. During the past month, how often did you eat pizza? Include frozen pizza, fast food pizza, and homemade pizza.

❍ Never
❍ 1 time last month
❍ 2–3 times last month
❍ 1 time per week
❍ 2 times per week
❍ 3–4 times per week
❍ 5–6 times per week
❍ 1 time per day
❍ 2 or more times per day

20. During the past month, how often did you have tomato sauces such as with spaghetti or noodles, or mixed into foods such as lasagna? Do NOT include tomato sauce on pizza.

❍ Never
❍ 1 time last month
❍ 2–3 times last month
❍ 1 time per week
❍ 2 times per week
❍ 3–4 times per week
❍ 5–6 times per week
❍ 1 time per day
❍ 2 or more times per day

21. Which of the following items were available in your home in the last week?
  	**Yes**	**No**
  Candy	❍	❍
  Cookies	❍	❍
  Potato chips or similar snack chip	❍	❍
  Ginger ale	❍	❍
  Regular cola or soda	❍	❍
  Diet soda	❍	❍
22. What is your gender?

❍ Male
❍ Female

23. What is your approximate age?

❍ 18–27
❍ 28–37
❍ 38–47
❍ 48–57
❍ 58–67
❍ 68–77
❍ 78 and above

24. What is your race? (Check all that apply)

❑ White
❑ Black or African American
❑ American Indian or Alaska Native
❑ Asian Indian
❑ Chinese
❑ Filipino
❑ Japanese
❑ Korean
❑ Vietnamese
❑ Other Asian
❑ Hawaiian Native
❑ Other Pacific Islander
❑ Some other race
❑ Choose not to provide

25. Are you of Spanish, Hispanic, or Latino origin?

❍ No
❍ Yes, Mexican, Mexican American
❍ Yes, Puerto Rican
❍ Yes, Cuban
❍ Yes, Other
❍ Choose not to provide

26. What is your height (in feet and inches)?

Feet
Inches

27. What is your approximate weight (in lbs)?

______ Weight in Pounds

28. Within the past 12 months I worried whether our food would run out before we got money to buy more.

❍ Often true
❍ Sometimes true
❍ Never true
❍ Don’t know or refuse

29. Within the past 12 months the food we bought just didn’t last and we didn’t have money to get more.

❍ Often true
❍ Sometimes true
❍ Never true
❍ Don’t know or refuse

30. Approximately how long have you received SNAP benefits? (Note: There is no limit for how long you can be on SNAP)

❍ Less than 1 year
❍ 1–2 years
❍ 2–3 years
❍ 3–4 years
❍ 4–5 years
❍ 5+ years

31. Would you say that in general your health is:

❍ Excellent
❍ Very Good
❍ Good
❍ Fair
❍ Poor
❍ Don’t know/Not sure
❍ Refuse to answer

32. Have you ever been told by a doctor or other health professional that you have any of the following conditions:
  	**Yes**	**No**	**Unsure**
  Diabetes or High blood sugar	❍	❍	❍
  High blood pressure	❍	❍	❍
  Overweight	❍	❍	❍
33. Please provide the following information, which is required to validate a winning voucher.
34. Your first initial:
35. Your last initial:
36. Last 4 digits of your EBT card:

Go to the next page to enter the lottery and see what you’ve won. We thank you again for taking this survey.

[*Next Page content: Only one pair, out of the following three pairs of messages, was displayed to the participant:*]

Congratulations! You have won one free reusable grocery bag this month! Even though you didn’t win an additional financial incentive, don’t forget that everyone at your farmers market-including you-receives an extra {IncentiveLevel} to spend on fruits and vegetables for every $1.00 in SNAP benefits that they redeem at the market. Standard SNAP incentive program guidelines and limits will apply.

Would you like to participate in this survey again next month?

❍ Yes
❍ No, please remove me from any future surveys.

Although you didn’t win an extra incentive this time, don’t forget that everyone at your farmers market-including you-receives an extra {IncentiveLevel} to spend on fruits and vegetables for every $1.00 in SNAP benefits that they redeem at the market. Standard SNAP incentive program guidelines and limits will apply.

Would you like to participate in this survey again next month?

❍ Yes
❍ No, please remove me from any future surveys.

Congratulations! You have won an extra incentive at your farmers market. You will receive an extra {IncentiveLevel} to spend on fruits and vegetables for every $1.00 in SNAP benefits that you redeem at the market. This voucher allows for unlimited matching (i.e., no daily or weekly limits on incentives) of the EBT dollars you have available to spend at the market.

Click the Next button below for instructions on how to redeem your voucher.

EXPIRES AFTER: {ExpirationDate} VALID ONLY AT: {RegionalFarmersMarket}

Would you like to participate in this survey again next month?

❍ Yes
❍ No, please remove me from any future surveys.

## Appendix B

Hispanic/Latino Survey Item (adapted from the 2020 U.S. Census)

Hispanic ethnicity categories can be reported descriptively by origin group and collapsed into a categorical variable for further analysis (e.g., Mexican, Puerto Rican, Cuban, Central American, and South American).

Are you of Hispanic, Latino, or Spanish origin?

❍ No
❍ Yes, Mexican, Mexican-American, Chicano
❍ Yes, Puerto Rican
❍ Yes, Cuban
❍ Yes, Salvadoran
❍ Yes, Dominican (Dominican Republic)
❍ Yes, Another Hispanic, Latino, or Spanish origin—*Please enter below*

*(for example, Colombian, Guatemalan, Spaniard, Ecuadorian, etc.)*
______________

❍ Choose not to provide
